# *Plasmodium falciparum* resistant to artemisinin and diagnostics have emerged in Ethiopia

**DOI:** 10.1038/s41564-023-01461-4

**Published:** 2023-08-28

**Authors:** Abebe A. Fola, Sindew M. Feleke, Hussein Mohammed, Bokretsion G. Brhane, Christopher M. Hennelly, Ashenafi Assefa, Rebecca M. Crudal, Emily Reichert, Jonathan J. Juliano, Jane Cunningham, Hassen Mamo, Hiwot Solomon, Geremew Tasew, Beyene Petros, Jonathan B. Parr, Jeffrey A. Bailey

**Affiliations:** 1 Center for Computational Molecular Biology, Brown University, Providence, RI USA; 2 Department of Pathology and Laboratory Medicine, Warren Alpert Medical School, Brown University, Providence, RI USA; 3 Ethiopian Public Health Institute, Addis Ababa, Ethiopia; 4 Institute for Global Health and Infectious Diseases, University of North Carolina, Chapel Hill, NC USA; 5 Harvard T. H. Chan School of Public Health, Harvard University, Boston, MA USA; 6 Global Malaria Programme, World Health Organization, Geneva, Switzerland; 7 Department of Microbial, Cellular and Molecular Biology, College of Natural and Computational Sciences, Addis Ababa University, Addis Ababa, Ethiopia; 8 Federal Ministry of Health, Addis Ababa, Ethiopia

**Keywords:** Parasite genomics, Infectious-disease diagnostics

## Abstract

Diagnosis and treatment of *Plasmodium falciparum* infections are required for effective malaria control and are pre-requisites for malaria elimination efforts; hence we need to monitor emergence, evolution and spread of drug- and diagnostics-resistant parasites. We deep sequenced key drug-resistance mutations and 1,832 SNPs in the parasite genomes of 609 malaria cases collected during a diagnostic-resistance surveillance study in Ethiopia. We found that 8.0% (95% CI 7.0–9.0) of malaria cases were caused by *P. falciparum* carrying the candidate artemisinin partial-resistance *kelch13* (*K13*) 622I mutation, which was less common in diagnostic-resistant parasites mediated by histidine-rich proteins 2 and 3 (*pfhrp2/3*) deletions than in wild-type parasites (*P* = 0.03). Identity-by-descent analyses showed that *K13* 622I parasites were significantly more related to each other than to wild type (*P* < 0.001), consistent with recent expansion and spread of this mutation. *Pfhrp2/3-*deleted parasites were also highly related, with evidence of clonal transmissions at the district level. Of concern, 8.2% of *K13* 622I parasites also carried the *pfhrp2/3* deletions. Close monitoring of the spread of combined drug- and diagnostic-resistant parasites is needed.

## Abstract (web summary)

*Plasmodium falciparum* candidate artemisinin partial-resistance *Kelch13* 622I mutation co-occurs with *pfhrp2/3* deletions in Ethiopia.

## Main

Despite intensified control efforts, progress towards malaria elimination has stalled in recent years. *Plasmodium falciparum* malaria remains an overwhelming problem in Africa, where approximately 90% of global cases and deaths occur^1^. The World Health Organization (WHO) recommends artemisinin-combination therapies (ACTs), such as artemether-lumefantrine (AL) or artesunate-amodiaquine (AS-AQ), as the first-line treatments for uncomplicated *P. falciparum* malaria^2^. However, the malaria parasite has evolved drug resistance to most available antimalarial drugs^3,4^ and resistant strains emerge and rapidly spread^5,6^. Since 2008, *P. falciparum* parasites resistant to first-line ACTs have emerged in Southeast Asia^7,8^ and have spread to neighbouring regions^9,10^.

Research carried out in Africa has reported reduced efficacy of artemisinin, with slowed clearance times and increased recrudescences^11–14^. Mutations in the *kelch13* (*K13*) gene associated with partial resistance to artemisinins have been reported in Uganda, Tanzania and Rwanda^15–17^. In addition, parasites undetectable by widely used *P. falciparum* rapid diagnostic tests (RDTs) owing to deletion mutations of the histidine-rich proteins 2 and 3 (*pfhrp2/3*) genes have emerged in the Horn of Africa^18–20^. In Ethiopia, RDTs have been used since 2004 and more than 70% of cases are diagnosed by RDT^18^. Together, these mutations threaten both components of existing test-and-treat programmes because co-occurrence of *pfhrp2/3* deletions and *K13* mutations would yield parasites resistant to both diagnosis and treatment. Improved understanding of how these mutations emerge, interact and spread is critical to the success of future malaria control and elimination efforts across Africa.

In Ethiopia, the overall incidence of malaria is low, but the disease remains endemic in 75% of the country, with 65% of the population at risk^21^. More than 5 million episodes of malaria occur each year, and transmission is highly heterogeneous and seasonal^22^. The goal for malaria elimination in Ethiopia is 2030, and prompt diagnosis and treatment with efficacious drugs is a cornerstone of the malaria elimination programme^23^. The ACT AL has been a first-line treatment for uncomplicated falciparum malaria throughout Ethiopia since 2004 (ref. ^24^). AL remains highly efficacious^25^ but detection of the candidate artemisinin resistance *K13* 622I mutation in northern Ethiopia^26,27^ and high prevalence of residual submicroscopic parasitemia after ACT treatment have raised concern^12,25^. Documenting ACT usage and effectiveness is challenging due to notable levels of empiric treatment and poor adherence to full regimens. Before the ACT era, sulfadoxine-pyramethamine (SP) served as first-line therapy from 1998–2004 after replacing chloroquine, which continues to be used extensively for *Plasmodium vivax* treatment^24^.

To our knowledge, there are no published studies addressing either the prevalence of drug-resistance mutations among *pfhrp2/3-*deleted versus non-deleted strains, or their transmission patterns. We sought to bridge this knowledge gap with a comparative genomic analysis of drug resistance among *pfhrp2/3-*deleted and non-deleted parasites collected across three regions of Ethiopia. Using molecular inversion probe (MIP) sequencing for highly multiplexed targeted genotyping^28,29^, we assessed the prevalence of key drug-resistance mutations in three regions and checked for co-occurrence with *pfhrp2/3* deletion in Ethiopia.

## Results

### Complexity of infections estimation

**Extended Data Fig. 1:**
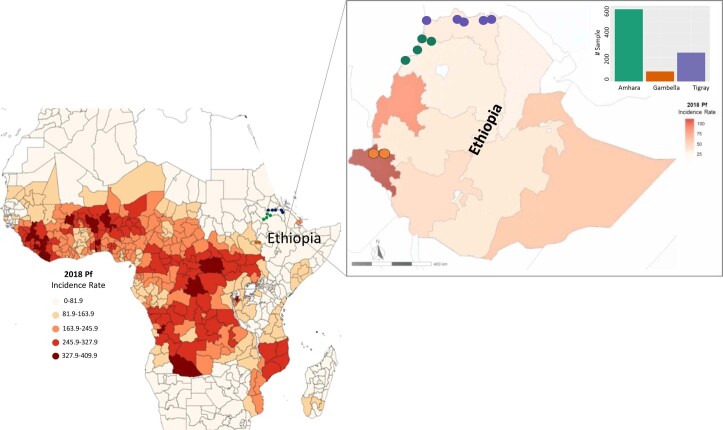
*Plasmodium falciparum* incidence rate in 2018 and distribution of sequenced samples (n = 609). Colors in the heat map indicate *P. falciparum* incidence rate per thousand cases in Africa year 2018. Zoomed Ethiopian map shows spatial distribution of sequenced samples at district level (colour dots in map) and regional level (color bar plot) and heat map indicate *P. falciparum* incidence rate per thousand cases in 2018 at regional level. Data source for this figure (https://data.malariaatlas.org)^53^.

**Extended Data Fig. 2:**
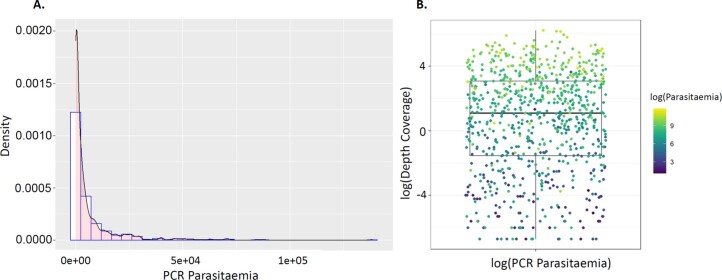
PCR parasitemia distribution and association between sequencing coverage and parasitaemia. **A**) Density plot showing parasitemia distribution with median parasitemia = 1411 parasite/ul for all successfully sequenced samples (n = 609). **B**) Association between sequencing coverage and parasitaemia. The MIP sequencing success is parasitaemia dependent as shown in the heatmap color. n represents the number of samples used in each panel. The boxplot centre lines in B, show the median value, the upper and lower bounds show the 25th and 75th quantiles, respectively, and the upper and lower whiskers show the largest and smallest values, respectively.

**Extended Data Fig. 3:**
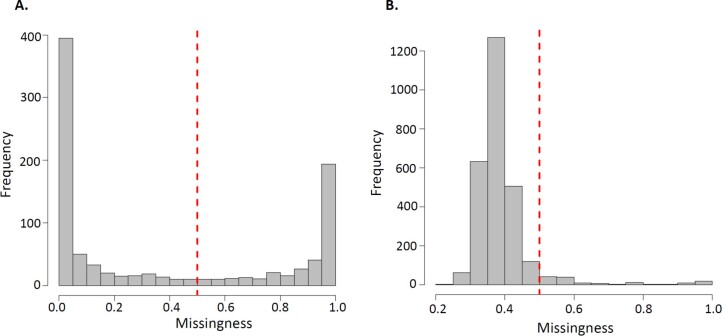
Sample and SNP missingness across sequenced samples using genome-wide MIP panel. **A**) Samples with >50% low-coverage loci were dropped as shown broken read line. **B**) Variant sites were then assessed by the same means in terms of the proportion of low-coverage samples, and sites with >50% low-coverage samples were dropped. Broken read line shows 50% threshold criteria we used to remove samples and loci from downstream analyses.

**Extended Data Fig. 4:**
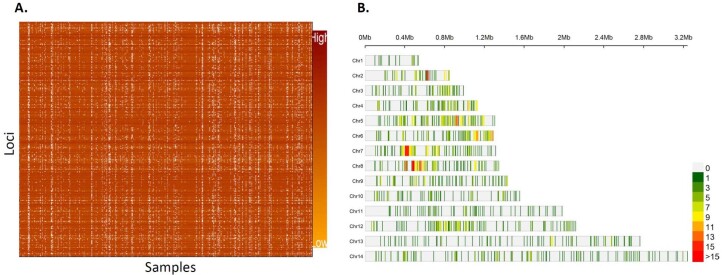
Successfully sequenced samples across three regions in Ethiopia and retained genome wide loci. **A**) Heatmap color shows samples (n = 609, columns) and loci (n = 1395, rows) coverage retained after filtering (Extended Data Fig. 3) for downstream analysis. **B**) Distribution of retained SNPs across *Plasmodium falciparum* chromosomes. The plot shows distribution of 1395 retained high quality biallelic SNPs across the 14 *P. falciparum* chromosomes within 0.025 Mb window size. Color coded from light grey for masked regions with no SNPs to red for regions containing high number SNPs per chromosome.

A total of 920 samples previously genotyped and MIP sequenced for *pfhrp2/3* deletions from three regions of Ethiopia (Amhara = 598, Gambella = 83, Tigray = 239) (Extended Data Fig. 1) were included in this analysis, representing dried blood spots (DBS) taken from a subset of the overall series of 2,637 malaria cases (Amhara = 1,336, Gambella = 622, Tigray = 679) (Supplementary Table 1). Samples had been collected from rural areas in 11 districts as part of a large *pfhrp2/3* deletion survey of 12,572 study participants (56% male, 44% female, age range 0–99 years) presenting with clinical signs and symptoms of malaria^18^. The districts were selected along the northwestern and western borders with Eritrea, Sudan and South Sudan as previously described^18^ (Extended Data Fig. 1). For this study, all samples were further MIP captured and sequenced using both a drug-resistance panel comprising 814 probes designed to target mutations and genes associated with antimalarial resistance and a genome-wide SNP panel comprising 1,832 probes designed for assessment of parasite relatedness and connectivity (Supplementary Data 1 and 2). Parasite densities across samples ranged from 3 to 138,447 parasites per µl, with median parasitaemia of 1,411 parasites per µl (Extended Data Fig. 2a); as expected, MIP sequencing coverage was parasite density-dependent (Extended Data Fig. 2b). All resistance genotypes with sufficient depth and quality were included in downstream analysis. After filtering for sample missingness and removing loci with low coverage (Extended Data Fig. 3), 609 samples and 1,395 SNPs from the genome-wide panel (Extended Data Fig. 4, and Supplementary Data 3 and 4) were included in downstream relatedness analysis.

**Extended Data Fig. 5:**
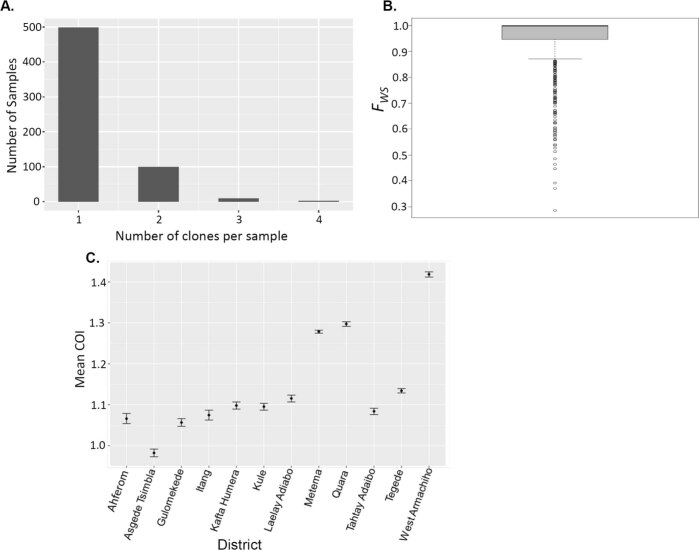
Complexity of infections. **A**) Distribution Number of clones per sample across all genotyped samples (n = 609) showing most of isolates carrying one clone (COI = 1). **B**) Cumulative within-infection FWS fixation showing majority of isolates classified as monogenomic (FWS > 0.95). Number of clones per sample. **C**) Spatial heterogeneity of mean complexity of infections per district across three regions in Ethiopia. Vertical lines show 95% confidence intervals. Samples size per district ranges 9–167 (see supplementary table 1).

Using filtered genome-wide SNPs, we calculated complexity of infection (COI) and adjusted for the relative proportion of DBS sampled from participants with discordant vs concordant RDT results since the parent *pfhrp2/3* survey purposefully oversampled the former. We estimate that the majority (82.4%, 95% confidence interval (CI) 80.7–83.6) of cases are monogenomic infections (COI = 1) (Extended Data Fig. 5 and Supplementary Table 1), reflecting relatively low ongoing transmission in the study areas. Overall, COI per sample ranged from 1 to 4 with variability at the district level (Extended Data Fig. 5c), consistent with heterogeneous malaria transmission at local scale.

### *K13* 622I mutation is prevalent in Ethiopia

**Fig. 1:**
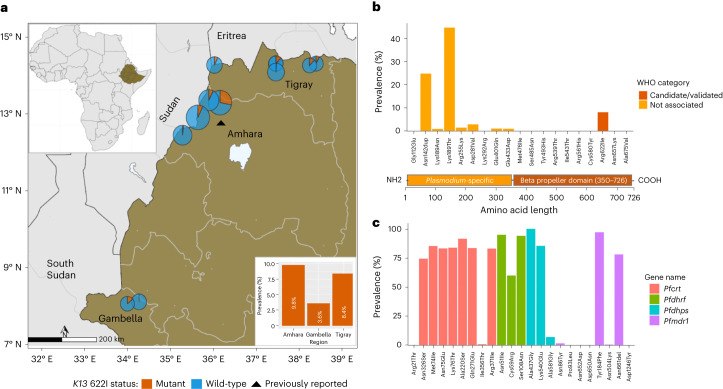
Prevalence of *K13* and key drug-resistance mutations in Ethiopia. **a**, Spatial distribution of *K13* 622I mutation at the district (pie charts) and regional (bar plot) levels. Colours indicate mutation status and pie chart size is proportional to sample size per district. The black triangle indicates the location where *K13* 622I mutation was reported previously. **b**, Prevalence of non-synonymous mutations across the *K13* gene, coloured according to WHO ACT resistance marker category. *K13* gene annotation shows 1–350 amino-acid residues in the poorly conserved *Plasmodium-*specific region and 350–726 residues in the beta propeller domain where validated resistance mutations are located. **c**, Prevalence of mutations across four key *P. falciparum* genes (colours) associated with commonly used antimalarial drugs.

Analysis of the drug-resistance markers revealed a high prevalence (8.0%, 95% CI 7.0–9.0) of samples expected to carry the WHO candidate artemisinin partial-resistance mutation 622I within the propeller domain of *K13*. The 622I mutation had only been previously described in Africa at a single site in Amhara, Ethiopia, near the Sudan border in 2014 at 2.4% prevalence^26^. Our results confirmed parasites with 622I in all 3 regions surveyed as well as all 12 districts (Fig. 1a). Highest prevalence was observed in Amhara (9.8%, 95% CI 8.2–11.4) in the northwest near the Sudan border, followed by Tigray (8.4%, 95% CI 6.2–10.5) near the Eritrea border and Gambella (3.6%, 95% CI 2.1–4.8) bordering South Sudan. However, there was high spatial heterogeneity at the district level and within regions (Supplementary Table 1). An additional 8 non-synonymous mutations were identified across the *K13* gene at low frequencies (<3%) except for K189T (44.4%), which is frequently observed in Africa and not associated with resistance (Fig. 1b). None of the other mutations were WHO-validated or candidate artemisinin partial-resistance mutations, and only two (*K13* E401Q and E433D) fell within the propeller region (Fig. 1b bottom panel, and Supplementary Table 1). To gain insight into relative fitness of 622I, we compared within-sample allele proportions in mixed mutant and wild-type infections (*n* = 16). On average, wild-type parasites occurred at relatively higher proportions (mean = 0.59) compared with 622I mutant parasites (mean = 0.41) (Mann–Whitney *P* = 0.025) in participants infected by more than one strain, suggesting lower fitness of mutant strains. The power of this analysis was limited as polygenomic infections were rare in this study but is consistent with competitive blood stage fitness costs.

### Prevalence of mutations that may augment ACT resistance

**Fig. 2:**
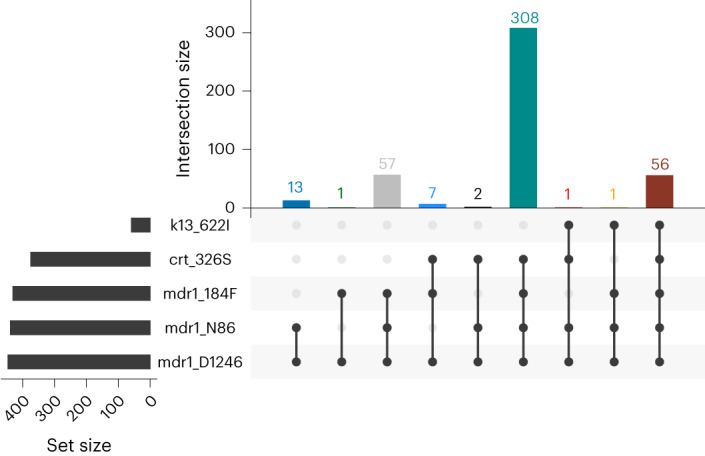
Frequency of key drug-resistance mutation combinations. The number of times (top right) each combination of mutations (bottom right) was observed is displayed, including *K13* 622I, *pfmdr1* N86 (wild), 184F (mutant) and D1246 (wild); and *pfcrt* genes. Only samples (*n* = 446) with complete genotypes across all loci representing monogenomic or the dominant haplotype in polygenomic infections are shown.

**Extended Data Fig. 6:**
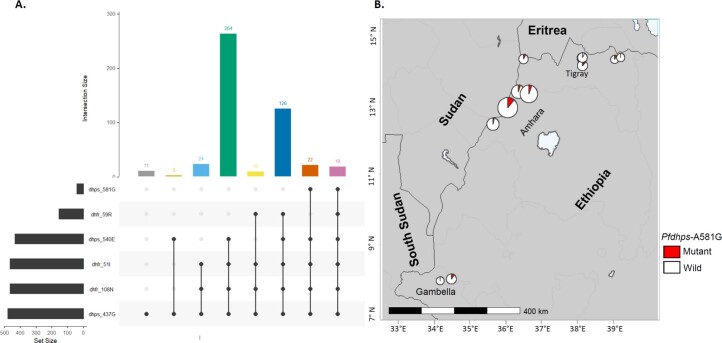
Prevalence of *Pfdhfr* and *Pfdhps* mutations across three regions in Ethiopia. **A**) UpSet plots showing the number of times each combination of mutations was seen for *Pfdhfr* and *Pfdhps*. **B**) Spatial distribution of *Pfdhps A581G* mutation at district level. Colors indicate mutation status and size of pie chart is proportional to sample size per district.

In addition to *K13* mutations, we found a number of key mutations in other *P. falciparum* genes associated with resistance to different antimalarial drugs (Fig. 1c and Supplementary Table 2), including ACT partner drugs. Mutations in the *P. falciparum* multidrug resistance gene 1 (*pfmdr1*), particularly isolates that carry the NFD haplotype (N86Y (wild), Y184F (mutant) and D1246Y (wild)), have been associated with decreased sensitivity to lumefantrine^30^. Overall, 83% of samples carry the NFD haplotype (Fig. 2) and 98% (60/61) of 622I mutant parasites carry *pfmdr1* NFD haplotypes. Although this difference was not significant (Fisher’s exact *P* = 0.34), the presence of 622I mutant parasites with *pfmdr1* NFD haplotypes raises questions about how the genetic background of 622I influences ACT efficacy in Ethiopia. We also investigated other mutations previously identified as backbone loci on which artemisinin partial-resistance-associated *K13* mutations are most likely to arise or could augment ACT resistance^31^. No parasites sampled in this study carried such background mutations (*pffd*-D193Y, *pfcrt*-I356T, *pfarps*-V127M and *pfmdr2*-T484I), except for *pfcrt*-N326S, which is carried by 98% of *K13* 622I and 81% of wild-type parasites (Fisher’s exact *P* < 0.001) (Fig. 2). The co-occurrences of 622I with the *pfmdr1* NFD haplotype and *pfcrt-*N326S raise concern about the efficacy of both artemisinin and partner drugs such as lumefantrine in Ethiopia. We also observed drug-resistance mutations in other genes (Supplementary Table 3), with high prevalence and some spatial heterogeneity in the distribution of mutations associated with SP resistance (Extended Data Fig. 6).

### Co-occurrence of drug-resistance and *pfhrp2/3* deletions

**Fig. 3:**
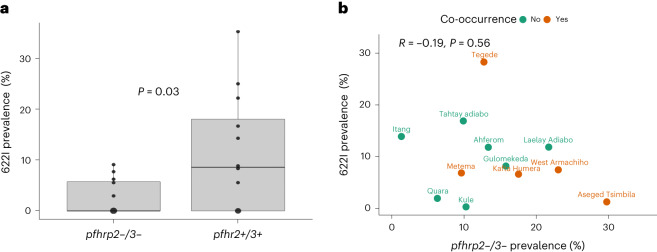
*K13* 622I mutation among *pfhrp2/3*-deleted and non*-*deleted parasite populations. **a**, Comparison of mean *K13* 622I mutation prevalence (*P* = 0.03, unpaired Student’s *t*-test, two-tailed) between *pfhrp2/3* double (*n* = 119) and *pfhrp2/3* non-deleted (*n* = 223) parasite populations by district across three regions in Ethiopia. **b**, Relationship between *pfhrp2/3* double*-*deleted parasite prevalence and *K13* 622I mutation prevalence by district. Prevalence estimates are weighted (see Supplementary Table 2). Orange points represent districts where parasites harbouring both *pfhrp2/3* deletions and *K13* 622I mutations are observed. The boxplot centre lines in **a** show the median value, the upper and lower bounds show the 25th and 75th quantiles, respectively, and the upper and lower whiskers show the largest and smallest values, respectively.

Overall, the *K13* 622I mutation is more common among *pfhrp2/3* non-deleted parasites (26/223, 11.6%) than among *pfhrp2/3* double*-*deleted parasites (5/110, 4.5%), although not significantly (Fisher’s exact *P* = 0.07). However, higher mean prevalence of the 622I mutation is observed among *pfhrp2/3* non-deleted parasites at the district level (unpaired Student’s *t*-test, two-tailed, *P* = 0.03) (Fig. 3a), which could be consistent with deleterious effects from the combination and/or independent origins with slow intermixing. We repeated this analysis using permutation by randomly reassigning double- and non-deleted groups and took the mean difference of these new groups. The permutation analysis shows −8.7% mean difference (*F*-statistic *P* = 0.02) in prevalence of 622I among *pfhrp2/3-*deleted vs non-deleted parasites, suggesting that patients infected by double-deleted parasites are more likely misdiagnosed and less likely received ACTs according to the country’s test-and-treat policy, which results in less ACT drug pressure. We observed a negative correlation between these mutations at the level of the individual collection sites, suggesting that different sites generally harbour one mutation or the other at high frequency. However, we observed a small number (*n* = 5) of parasites with both 622I mutation and *pfhrp2/3* deletion in sites where mutation or deletion frequency is high (Fig. 3b), confirming that recombination between parasites with these mutations is possible. Interestingly, 622I is more common among *pfhrp3-*deleted parasites (29/169, 17.2%) than among wild-type *pfhrp2/3* non-deleted parasites (26/223, 11.2%), but the difference was not statistically significant (chi-square *P* = 0.23).

**Extended Data Fig. 7:**
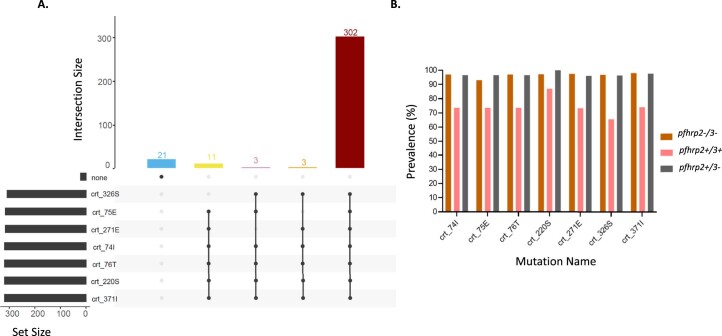
Prevalence of *Pfcrt* mutations across three regions in Ethiopia. The UpSet plot shows the number of times each combination of mutation was observed within *pfcrt* (**A**), and prevalence of these mutations by *pfhrp2/3* status (**B**). Note that the prevalence within *pfhrp2-/3+* was not estimated due to small sample size.

We also examined co-occurrence of *pfhrp2/3* deletions and other drug-resistance mutations, particularly *pfcrt* mutations, as most *pfhrp2/3* deletion reports so far have emerged in areas where *P. vivax* and *P. falciparum* are sympatric and chloroquine is used to treat vivax malaria^32^. We observed overall high prevalence (median 84% across districts) of *pfcrt* mutations (codon 74–76) (Extended Data Fig. 7a). The prevalence of *pfcrt-*K76T mutation was greater among *pfhrp2/3* double-deleted (96.3%) than among non-deleted (73.8%) parasites, but the difference was not statistically significant (chi-square *P* = 0.15, Extended Data Fig. 7b). This finding suggests that patients infected by *pfhrp2/3* double-deleted parasites may be more often exposed to chloroquine.

### Population structure of *K13* 622I and *pfhrp2/3-*deleted parasites

**Fig. 4:**
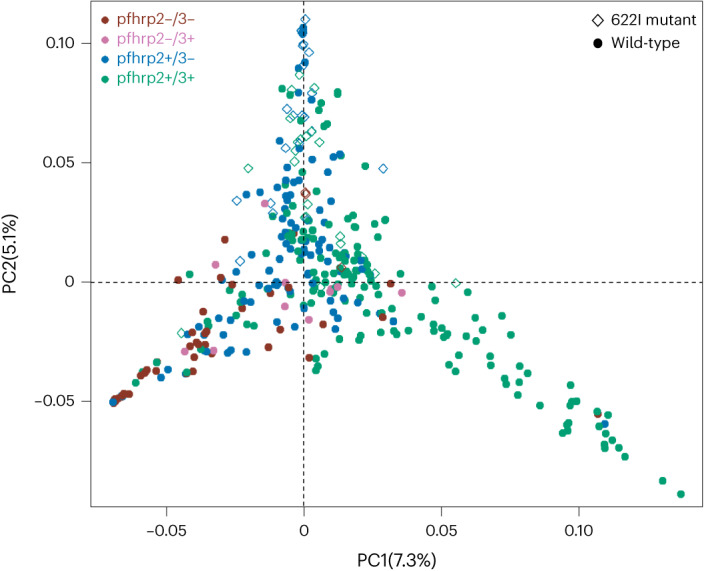
PCA of *P. falciparum* populations annotated by *K13* 622I and *pfhrp2/3* deletion genotypes. Colours indicate *pfhrp2/3* deletion status and shape indicates *K13* 622I mutation status. The percentage of variance explained by each principal component is presented.

**Extended Data Fig. 8:**
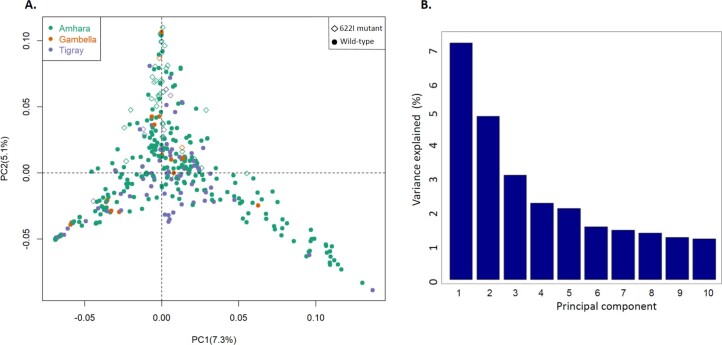
Population structure of *P. falciparum* in Ethiopia. **A**) Principal component analysis *P. falciparum* populations per region. Colors indicate sample origin and shape indicates *K13* 622I mutation status (circle indicates wild and diamond indicates mutant). Percentage of variance explained by each principal component presented (%). **B**) Percent of overall variance explained by the first 10 principal components during PCA.

**Extended Data Fig. 9:**
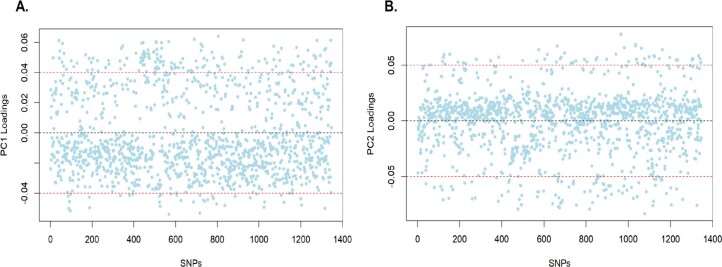
PCA loading values. PC1 (**A**) and PC2 (**B**) are shown by SNP. Cutoffs show SNPs that highly contribute to positive or negative distribution of samples in the PC plots.

We investigated genetic population structure using principal component analysis (PCA), which revealed clustering of parasites by *K13* 622I mutation (PC1) and by *pfhrp2/3* deletion (PC2) status, but not by geography (Fig. 4 and Extended Data Fig. 8a). Overall, 13.4% of variation in our dataset was explained by these first two principal components (Extended Data Fig. 8b). Analysis of loading values did not reveal SNPs or genomic regions with disproportionate influence on the observed population structure (Extended Data Fig. 9). Genetic differentiation between populations is low overall (*F*_st_ range = 0.002–0.008), lowest between Amhara and Tigray regions (*F*_st_ = 0.002), and highest between Gambella and Tigray regions (*F*_st_ = 0.008), followed by between Amhara and Gambella (*F*_st_ = 0.003).

### Genetic relatedness of *K13* 622I and *pfhrp2/3-*deleted parasites

**Fig. 5:**
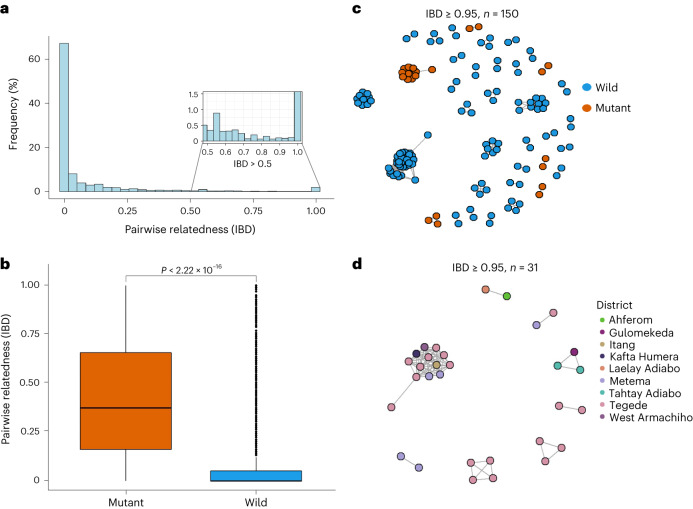
Pairwise IBD sharing and relatedness networks suggest clonal transmission and expansion of *K13* 622I parasites. **a**, Pairwise IBD sharing across all three regions of Ethiopia. The plot shows the probability that any two isolates are identical by descent, where the *x* axis indicates IBD values ranging 0–1 and the *y* axis indicates the frequency (%) of isolates sharing IBD. The inset highlights highly related parasite pairs from out of total pairs (*n* = 44,883), with a heavy tail in the distribution and some highly related pairs of samples having IBD ≥ 0.95. **b**, Pairwise IBD sharing within parasites carrying *K13* 622I vs wild type (*P* < 0.001, two-tailed, Mann–Whitney *U*-test). Boxes indicate the interquartile range, the line indicates the median, the whiskers show the 95% confidence intervals and black dots show outlier values. *P* value determined using Mann–Whitney test is shown. **c**, Relatedness network of highly related parasite pairs (*n* = 150) sharing IBD ≥ 0.95. Colours correspond to *K13* 622I mutant and wild parasites. **d**, Relatedness network of only *K13* 622I parasite pairs (*n* = 31) sharing IBD ≥ 0.95 at the district level/local scale. Colours correspond to districts across three regions in Ethiopia. In both **c** and **d**, each node identifies a unique isolate and an edge is drawn between two isolates if they share their genome above IBD ≥ 0.95. Isolates that do not share IBD ≥ 0.95 of their genome with any other isolates are not shown.

Identity-by-descent (IBD) analysis revealed evidence of recent clonal transmission and spread of *K13* 622I parasites. Overall, 10.6% of pairs (4,758 pairs out of 44,883) are highly related (IBD ≥ 0.25, half siblings) (Fig. 5a). We observe a tailed distribution of highly related parasite pairs, with 26.6% of pairwise comparisons sharing their genome at an IBD value of ≥0.05. Comparing *K13* 622I mutant and wild-type parasites, we find significantly higher mean pairwise IBD sharing within *K13* 622I mutant populations (0.43 vs 0.08, respectively; Mann–Whitney *P* < 0.001) (Fig. 5b). Network analysis of highly related parasites (pairwise IBD ≥ 0.95) shows that 622I mutant parasites tend to form related clusters and pairs separate from wild-type parasites (Fig. 5c), consistent with clonal transmissions of 622I parasite populations in Ethiopia. The majority of clonal parasites carrying the 622I mutation originated from one district (Tegede) (Fig. 5d), probably illustrating an outbreak with rapid spread (Supplementary Table 1).

**Fig. 6:**
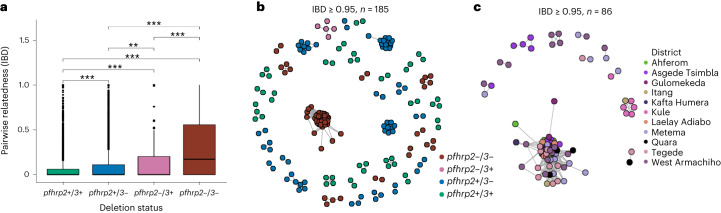
Pairwise IBD sharing and relatedness networks suggest independent emergence and clonal spread of *pfhrp2/3*-deleted parasites. **a**, Pairwise IBD sharing by *pfhrp2/3* deletion status (***P < 0.001, **P < 0.01, Kruskal–Wallis test). Boxes indicate the interquartile range, the line indicates the median, the whiskers show the 95% confidence intervals and black dots show outlier values. **b**, Relatedness network of highly related parasite pairs sharing IBD ≥ 0.95. Each node identifies a unique isolate and an edge is drawn between two isolates if they share their genome at IBD ≥ 0.95. Isolates that do not share IBD ≥ 0.95 of their genome with any other isolates are not shown. Colour codes correspond to *pfhrp2/3* deletion status. **c**, Relatedness network of *pfhrp2/3* double-deleted parasite pairs with IBD ≥ 0.95 at district level/local scale. Colours correspond to districts across three regions of Ethiopia.

*Pfhrp2/3-*deleted parasites also have higher relatedness than wild-type parasites, with significantly different pairwise IBD sharing (Kruskal–Wallis test *P* < 0.001) when comparing *pfhrp2/3* double-, single and non-deleted parasites (Fig. 6a). Pairwise IBD sharing is highest among *pfhrp2/3* double-deleted parasites, with 43.7% of comparisons having IBD ≥ 0.25 (half siblings), compared with only 4.3% of *pfhrp2/3* non-deleted parasites. Network analysis of highly related isolates (IBD ≥ 0.95) revealed clustering by deletion status (Fig. 6b), with district-level clustering of *pfhrp2/3* double-deleted parasites evident in Kule, Atse-Tshimbila and West-Armachiho (Fig. 6c), a finding consistent with clonal spread of *pfhrp2/3* double-deleted parasites at the local scale.

## Discussion

Our genetic analyses confirms that the WHO candidate artemisinin partial-resistance *kelch* 622I mutation is common in three regions of Ethiopia and suggests recent clonal spread of this mutation. We observed low levels of polyclonality in our study, consistent with previous study findings^24^, and relatively low to moderate malaria transmission intensity in these regions. Our findings suggest that independent transmission of highly related 622I or *pfhrp2/3-*deleted parasites predominates, with bursts of clonal spread. We propose that Ethiopia’s intensive test-and-treat strategies have exerted substantial selective pressure on the *P. falciparum* population and are driving rapid expansion of artemisinin- and diagnostic-resistant parasites. Although rare, identification of parasites carrying both 622I and *pfhrp2−/3−* deletions raises concern that parasites with partial resistance to treatment and the ability to escape HRP2-based RDT detection are circulating in Ethiopia.

Continued use of ACTs and other antimalarials puts pressure on the *P. falciparum* population and could be one factor driving the emergence of antimalarial drug resistance. ACTs have been the first-line treatment for uncomplicated falciparum malaria in Ethiopia for nearly two decades, with primaquine now recommended to interrupt transmission and oral quinine used for pregnant women during the first trimester. Parenteral artesunate (or quinine when it is unavailable) is the first-line treatment for severe malaria^23^. Chloroquine followed by radical cure with primaquine is recommended for patients with *P. vivax* malaria^24^. Thus, parasite populations are exposed to multiple antimalarial drugs.The presence of *K13* 622I across all sampled districts signals that parasites are under ACT pressure in Ethiopia and indicates that parasites are evolving to escape antimalarial treatment. The 622I mutation was reported previously in two small studies from one site in northern Ethiopia (Amhara region), with associated delay in parasite clearance on day 3 of ACT^26^ and increased prevalence over time, from 2.4% in 2014 (ref. ^26^) to 9.5% in 2017–2018 (ref. ^27^). While not yet peer reviewed, reports of 622I at high prevalence in Eritrea (16.7% in 2016) and association with 6.3% delayed clearance on day 3 of AL treatment raise further concern about this mutation^33^. The higher prevalence of the 622I mutation in northern Ethiopia (Amhara region) in our study suggests that it originated in northern Ethiopia or Eritrea, although our data are insufficient to determine its origins. The lower frequency of 622I vs wild-type parasites in polyclonal infections provides evidence that it may decrease fitness within the human host, a consistent trait of artemisinin partial-resistance mutations due to loss of function within the *K13* propeller. Taken together, these findings suggest that 622I in Ethiopia represents a meaningful threat to elimination efforts across the Horn of Africa.

As transmission is reduced in Ethiopia and other countries nearing elimination, the majority of infected individuals are expected to carry single rather than multiple parasite strains. Majority (82%) of genotyped samples in our study are monogenomic, consistent with previous findings^24^. The associated increased rate of inbreeding in such settings^34^ is known to favour the spread of drug-resistant strains^35,36^. Decreased parasite competition in low-transmission settings allows expansion of strains with resistance mutations that make them relatively less fit in the absence of drug pressure. This is the case for artemisinin partial resistance. We previously reported that false-negative HRP2-based RDT results owing to *pfhrp2/3* deletions are common in Ethiopia and that *pfhrp2* deletion is under recent positive selection^18^. Using a larger MIP panel targeting SNPs across the genome for IBD analysis, we now show that these parasites are closely related and that bursts of clonal transmission appear to be occurring at the district or local scale. These findings support the hypothesis that low transmission and associated parasite inbreeding are important for the expansion of *pfhrp2/3-*deleted populations. This is also consistent with the idea that outcrossing may disrupt co-transmission of *pfhrp2* and *pfhrp3* deletions given that they are on separate chromosomes.

The rare presence of parasites with both *K13* 622I and *pfhrp2/3*-deletion mutations is worrying. Their co-existence in a small number of parasites may simply be a consequence of their distinct origins and insufficient time for the expansion of 622I, *pfhrp2/3*-deleted parasite strains. While combined fitness costs may also have a role in the low prevalence of parasites with both mutations^16,37^, in the absence of inter-strain competition in low transmission settings, there may be few barriers to the spread of 622I, *pfhrp2/3-*deleted parasites. Our analysis of drug-resistance mutations and parasite population structure confirms that close monitoring of emerging drug- and diagnostic-resistant strains is urgently needed to inform control strategies in the Horn of Africa and neighbouring countries.

Other studies have suggested that high efficacy of partner drugs (that is, lumefantrine) can prevent the spread of ACT resistance in Africa^16,37^. However, we observe high prevalence of mutations associated with resistance to other antimalarial drugs in our study, with almost all genotyped samples carrying the ACT partner drug lumefantrine resistance haplotype (*pfmdr1* NFD)^38,39^ and more than 80% carrying the *pfcrt*-N326S background mutation that augments artemisinin partial resistance. No parasites sampled in this study carried other common background mutations observed in South-East Asia (*pffd*-D193Y, *pfcrt*-I356T, *pfarps*-V127M and *pfmdr2*-T484I)^31^. Together, these findings support the need for close monitoring of the efficacy of lumefantrine and other partner drugs across Ethiopia.

IBD sharing was higher within the *K13* 622I mutant parasite population compared with wild-type parasites, suggesting that the 622I mutation emerged or entered into northern Ethiopia in the recent past^27^ and spread to other parts of the country. Highly related parasites are also closely clustered at the district level, a finding expected after clonal transmission. Moreover, our finding of parasites with high IBD and low overall COI in this study indicates low ongoing transmission across the three regions and that most recombination is between highly related or clonal strains^40,41^. IBD analysis also showed high relatedness and clonal expansion of *pfhrp2/3* double-deleted parasites (most probably not detected by HRP2*-*based RDTs) at the local scale, with distinct populations of very closely related *pfhrp2/3-*deleted parasites observed in several districts. Clonal spread with local inbreeding could facilitate rapid spread of *pfhrp2/3-*deleted parasites that are expected to escape diagnosis by RDTs. Our data also reveal higher prevalence of the 622I mutation among *pfhrp2/3* non-deleted compared with double-deleted parasites, a finding that might be seen when *pfhrp2/3* deletion leads to misdiagnosis, leaves patients untreated and results in *pfhrp2/3-*deleted parasites exposed to less ACT pressure. Supporting this idea, we observed more frequent co-occurrence of *pfcrt-*K76T mutation and *pfhrp2/*3-deleted parasites suggestive of empirical chloroquine treatment for presumed non-falciparum malaria.

Our study has limitations. First, travel histories from malaria cases and samples from neighbouring countries are not included; hence tracking resistant-strain importation is not addressed in detail. Second, the parent study was designed to evaluate RDT failure and could introduce selection bias, including undersampling of low parasitaemia and submicroscopic infections, or oversampling of monogenomic infections. We therefore adjusted our *K13* 622I prevalence estimates to improve the generalizability of our findings. Third, the areas studied represent regions with relatively higher transmission (Amhara, Gambella and Tigray) and do not include other parts of the country, making it difficult to extrapolate our findings across the country. It may be that other regions have lower prevalences of drug- and diagnostic-resistance mutations, or that prevalences are even higher in lower-transmission settings. Further study within Ethiopia and surrounding countries is warranted.

Overall, our study suggests that the ongoing selective pressures exerted on parasite populations in Ethiopia by HRP2-based RDT diagnosis^42^ and ACT treatment^43^ might result in co-occurrence of diagnostic and drug resistance, representing a double threat to malaria elimination. However, Ethiopia’s recent transition to alternative RDTs might reduce selective pressures that favour *pfhrp2/3-*deleted strains. Evidence from South America, where RDTs have never been widely used but *pfhrp2/3* deletions are common, confirms that other factors beyond RDT diagnostic pressure are probably necessary for their emergence. As Ethiopia and other countries in the Horn of Africa approach malaria elimination, diagnostic and drug resistance may be more likely to co-occur.

Many sites in Africa are using targeted high-throughput sequencing strategies such as MIPs and multiplex amplicons for drug-resistance surveillance. In future, we expect genomic surveillance coupled with large-scale epidemiologic surveys to become the norm across Africa, providing an unprecedented view of emerging drug resistance in Africa that can inform control and elimination efforts.

## Methods

### Study sites and sample genotyping

A total of 920 samples from three regions (Amhara = 598, Gambella = 83 and Tigray = 239) (Extended Data Fig. 1) previously assessed for *pfhrp2/3* deletions^18^ were further genotyped using MIPs. Sampling strategy, sample collection, DBS sample transportation, DNA extraction and initial molecular analysis were described in detail in our previous study^18^. The parent study was approved by the Ethiopian Public Health Institute (Addis Ababa, Ethiopia; protocol EPHI-IRB-033-2017) and the World Health Organization Research Ethics Review Committee (Geneva, Switzerland; protocol ERC.0003174 001). Parasite sequencing and analysis of de-identified samples were deemed nonhuman subjects research by the University of North Carolina at Chapel Hill (North Carolina, USA; study 17-0155).

### MIP capture, sequencing and variant calling

DNA originally isolated from DBS samples was captured and sequenced using two separate MIP panels: (1) a drug-resistance panel (*n* = 814) designed to target mutations and genes associated with antimalarial resistance and (2) a genome-wide panel (*n* = 1,832) designed to target SNPs to evaluate parasite connectivity and relatedness (Supplementary Data 1 and 2)^28,29^. Details of company names and catalogue numbers for different reagents used for MIP capturing and sequencing are provided in Supplementary Data 1. MIP capture and library preparation were performed as previously described^17^. Sequencing was conducted using an Illumina NextSeq 550 instrument (150 bp paired-end reads) at Brown University (Rhode Island, USA).

The MIPtools (v.0.19.12.13; https://github.com/bailey-lab/MIPTools) bioinformatic pipeline was used for processing of sequencing data and variant calling. Briefly, this pipeline employs MIPWrangler software to stitch paired reads, remove sequence errors and predict MIP microhaplotypes, leveraging the unique molecular identifiers (UMIs) in each arm. The haplotypes for each target were mapped to the *P. falciparum* 3D7 reference genome (PlasmoDB-42_Pfalciparum3D7 obtained from https://plasmodb.org/plasmo/app) using Burrows–Wheeler Aligner (BWA)^44^ and variant calling was performed on these samples using freebayes^45^. Downstream analyses were performed on generated variant calling files (VCF) as well as translated tables based on 3D7 transcriptome for coding mutations. For the genome-wide MIP panel, variants were quality filtered by removing those with less than 3 UMIs within a sample and less than 10 UMIs across the entire population. The drug-resistance panel included known SNPs in *pfcrt*, *pfdhfr*, *pfdhps*, *pfmdr1*, *K13* and other putative drug-resistance genes and has been described elsewhere^28^ (Supplementary Data 2). Unweighted prevalence was calculated as (*p* = *m*/*n* × 100, where *p* is the prevalence, *m* is the number of infections with mutant alleles and *n* is the number of successfully genotyped infections) (Supplementary Table 3). Unweighted prevalence was calculated using the miplicorn R package v.0.2.90 (https://github.com/bailey-lab/miplicorn) and the vcfR R package v.1.13.0 (ref. ^46^). Mutant combinations were plotted and visualized using the ‘UpSet’ Package in R (v.1.4.0)^47^. Because dried blood spot sampling differed on the basis of RDT results (participants with HRP2−/PfLDH+ results were purposefully oversampled for molecular characterization in the parent study), we adjusted *K13* 622I and other key antimalarial drug-resistance mutations prevalence estimates by weighting for the relative sampling proportions of RDT-concordant (HRP2+) and discordant (HRP2−/PfLDH+) samples. This was achieved by weighting RDT profile-specific prevalence estimates by the total number of *P. falciparum-*positive individuals presenting with that RDT profile in the parent study by district, region and overall. Finally, 95% confidence intervals for these weighted prevalence estimates were estimated using bias-corrected and accelerated bootstrapping (*n* = 2,000 replications for district and region-level estimates, *n* = 3,000 replications for overall study estimate) using the R packages boot (v.1.3–28) and confintr (v.0.2.0). Mutant combinations were plotted and visualized using the ’UpSet’ Package in R (v.1.4.0)^47^. For the genome-wide MIP panel, only biallelic variant SNPs were retained for analysis. Genome positions with more than 50% missing data (Extended Data Fig. 3a) and samples missing 50% of sites (Extended Data Fig. 3b) were removed, leaving 609 samples and 1,395 SNPs from the genome-wide panel (Extended Data Fig. 4a), which are distributed across 14 *P. falciparum* chromosomes (Extended Data Fig. 4b). The drug-resistance panel includes SNPs across known *P. falciparum* drug-resistance genes that have been described elsewhere^28^.

### COI

To estimate the COI, we used THE REAL McCOIL R package categorical method^48^. As DBS sampling in the parent study favoured RDT discordant samples (HRP2−/pfLDH+) and could bias our COI estimates, we estimated overall and district-level prevalence of monogenomic infections by weighting for the relative sample proportions of RDT-concordant and discordant samples in the parent survey. The same approach (as mentioned above for 622I) was used to estimate weighted prevalence of monogenomic vs polygenomic infections at the district level. We also calculated the within-host fixation index (*F*_ws_) using the R package moimix (v.0.2.9)^49^, which measures the probability that any random pair of infections carries different alleles at a specific locus, as another measure of within-host diversity of the parasites. It was calculated for each infection as follows: *F*_ws_ = 1 − (*H*_w_/*H*_s_), where *H*_w_ is the infection heterozygosity across all loci and *H*_s_ is the heterozygosity of the population from which the infection was sampled. As *F*_ws_ calculation based on the frequency of alleles per individual relative to that within the source population, it allows comparison between populations. As *F*_ws_ values range from 0 to 1, the sample was classified as having multiple infections (polyclonal) if *F*_ws_ < 0.95 and monoclonal (single-strain) infections if *F*_ws_ ≥ 0.95. Samples with *F*_ws_ < 0.95 were considered to come from mixed strain infections, indicating within-host diversity.

### Population structure and genetic differentiation

To assess whether parasite populations within Ethiopia clustered on the basis of their geographic origin or their *pfhrp2/3* deletion status, we first conducted PCA using the SNPRelate R package (v.1.30.1)^50^. The eigenvalues generated from filtered VCF file using the snpgdsPCA function were used as input file for PCA and the resulting PCs were visualized using the ggplot2 R package (v.3.4.0). We calculated pairwise genetic differentiation (*F*_ST_) as a measure of genetic divergence between populations using the PopGenome R package (v.2.7.5)^51^.

### Analysis of parasite relatedness using IBD

To measure relatedness between *P. falciparum* parasites and identify regions of the genome shared with recent common ancestry, the inbreeding_mle function of the MIPAnalyzer software (v.1.0.0) was used on monogenomic samples to calculate IBD^29^. We determined IBD sharing variation at regional and local scale (district level) to assess spatial patterns of parasite connectivity and transmission dynamics at micro-local level, comparing deleted and mutant parasites vs wild-type parasites. Networks of highly related parasites per *K13* 622I mutation status or *pfhrp2/3* deletion status were generated using the igraph R package (v.1.3.5)^52^.

### Reporting summary

Further information on research design is available in the Nature Portfolio Reporting Summary linked to this article.

### Supplementary information

Supplementary Information Descriptions of Supplementary Tables 1–3 and Data 1–6.

Reporting Summary

Supplementary Tables 1–3 Weighted overall and district prevalences for monogenomic infections and key drug resistant mutations, as well as unweighted prevalences for all nonsynonymous mutations from drug resistance MIP panel.

Supplementary Data 1–4 Detailed target information, sample metadata, and sample-level genotypes for genome-wide and drug resistance MIP panels.

Supplementary Data 5 Genome-wide MIP panel filtered variant file.

Supplementary Data 6 Parasite (Pf) MIP capture 96-well protocol.

## Data Availability

All sequencing data are available under accession no. SAMN35531338-SAMN35530730 at the Sequence Read Archive (SRA) (http://www.ncbi.nlm.nih.gov/sra), and the associated BioProject is PRJNA978031. De-identified datasets generated during the current study and used to make all figures are available as supplementary files or tables.

## Code availability

Code used during data analysis is available on GitHub at https://github.com/Abefola/EPHI_622I_hrp23_project. Additional software packages and tools that are useful when working with MIP data are available at https://github.com/bailey-lab/MIPTools and https://github.com/Mrc-ide/mipanalyzer.
