# Supplementary material for: Plasmodium falciparum resistant to artemisinin and diagnostics have emerged in Ethiopia
Source: Nat Microbiol. 2023 Aug 28;8(10):1911–9. doi: 10.1038/s41564-023-01461-4 (PMC10522486; doi:10.1038/s41564-023-01461-4)
Supplement: Supplementary file 1 — Descriptions of Supplementary Tables 1–3 and Data 1–6. [file 41564_2023_1461_MOESM1_ESM.pdf]

nature microbiology

Analysis

https://doi.org/10.1038/s41564-023-01461-4

Plasmodium falciparum resistant to artemisinin and diagnostics have emerged in Ethiopia

In the format provided by the authors and unedited

Supplementary Information

I. Supplementary Tables

Supplementary tables are compiled into a single file (Bailey_SupplmentTables1-3 _ NMICROBIOL-23030538A.xlsx) for ease of viewing.

Supplementary Table 1. Supplementary Table 1. Prevalence of monogenomic infections per district weighted by RDT discordant prevalence. COI = complexity infection, n = number of samples successfully genotyped at district level.

Supplementary Table 2. Weighted prevalence of key drug resistance mutations per district. Weighting calculated by RDT profile (see Methods). COI = complexity infection, n = number of samples successfully genotyped at district level.

Supplementary Table 3. District level prevalence (unweighted) of all nonsynonymous mutations across different drug resistance genes. The prevalence of antimalarial resistance mutations detected by the MIPs is shown for each geographic location. Mutations above 1% prevalence in any of the districts shown. AA = Amino Acid; n = number of samples genotyped; m = number of samples carry mutant allele.

II. Supplementary Data

Supplementary tables (Bailey_SupplmentData1-4_ NMICROBIOL-23030538A.xlsx) are compiled into a single file for ease of viewing.

Supplementary Data 1. List of loci across *Plasmodium falciparum* genome included in genome wide MIP panel.

Supplementary Data 2. List of drug resistance markers across *Plasmodium falciparum* genome included in drug resistance MIP panel.

Supplementary Data 3. Metadata for 609 successfully sequenced samples using the genome wide MIP panel.

Supplementary Data 4. Metadata and genotype file for all successfully sequenced samples (varies per marker) using the drug resistance MIP panel. Only successfully sequenced samples were used for drug resistance marker prevalence estimates. 0 = Reference, Call, 1 = Alternative heterozygous call, 2= Alternative homozygous call, -1 = Missing call.

Supplementary Data 5. Genome-wide MIP panel filtered variant file.

Supplementary Data 6. *Plasmodium falciparum* MIP Capture 96-well Protocol.
