# Supplementary material for: Plasmodium falciparum resistant to artemisinin and diagnostics have emerged in Ethiopia
Source: Nat Microbiol. 2023 Aug 28;8(10):1911–9. doi: 10.1038/s41564-023-01461-4 (PMC10522486; doi:10.1038/s41564-023-01461-4)
Supplement: Supplementary file 2 — Reporting Summary [file 41564_2023_1461_MOESM2_ESM.pdf]

nature portfolio

Corresponding author(s): Jeffrey A. Bailey

Last updated by author(s): Jul 14, 2023

Reporting Summary

Nature Portfolio wishes to improve the reproducibility of the work that we publish. This form provides structure for consistency and transparency in reporting. For further information on Nature Portfolio policies, see our Editorial Policies and the Editorial Policy Checklist.

Statistics

For all statistical analyses, confirm that the following items are present in the figure legend, table legend, main text, or Methods section.

n/a	Confirmed	
☐	☒	The exact sample size (*n*) for each experimental group/condition, given as a discrete number and unit of measurement
☐	☒	A statement on whether measurements were taken from distinct samples or whether the same sample was measured repeatedly
☐	☒	The statistical test(s) used AND whether they are one- or two-sided<br>*Only common tests should be described solely by name; describe more complex techniques in the Methods section.*
☒	☐	A description of all covariates tested
☐	☒	A description of any assumptions or corrections, such as tests of normality and adjustment for multiple comparisons
☐	☒	A full description of the statistical parameters including central tendency (e.g. means) or other basic estimates (e.g. regression coefficient) AND variation (e.g. standard deviation) or associated estimates of uncertainty (e.g. confidence intervals)
☐	☒	For null hypothesis testing, the test statistic (e.g. *F*, *t*, *r*) with confidence intervals, effect sizes, degrees of freedom and *P* value noted<br>*Give P values as exact values whenever suitable.*
☒	☐	For Bayesian analysis, information on the choice of priors and Markov chain Monte Carlo settings
☒	☐	For hierarchical and complex designs, identification of the appropriate level for tests and full reporting of outcomes
☒	☐	Estimates of effect sizes (e.g. Cohen's *d*, Pearson's *r*), indicating how they were calculated

Our web collection on statistics for biologists contains articles on many of the points above.

Software and code

Policy information about availability of computer code

Data collection	In parent study, data was collected in the field using paper forms and double entered into Epi Info (v3.2). Discrepancies were cross-checked against the hard copy paper forms and resolved by consensus. No software was used for data collection in the current study.
Data analysis	MIP sequenced fastq file processing were performed using MIPTools (v0.19.12.13), which uses the MIPWrangler algorithm (v1.2.0), bwa (v0.7.17), and freebayes (v1.3.1). Prevalence was calculated using the miplicorn R package version 0.2.90 (https://github.com/bailey-lab/miplicorn) and vcfR R package version 1.13.0. A 95% confidence intervals prevalence estimates were estimated using bias corrected and accelerated (BCa) bootstrapping (n = 2000 replications for district and region-level estimates, n = 3000 replications for overall study estimate) using the R packages boot (version 1.3-28) and confintr (version 0.2.0). Final, mutant combinations were plotted and visualized using UpSet Package in R version 1.4.0. R package moimix (version 0.2.9) was used to calculated within-host fixation index (Fws). Code used during data analysis is available through GitHub at https://github.com/Abefola/EPHI_622I_hrp23_project. Additional software packages and tools that are useful when working with MIP data are available at https://github.com/bailey-lab/MIPTools and https://github.com/Mrc-ide/mipanalyzer.

For manuscripts utilizing custom algorithms or software that are central to the research but not yet described in published literature, software must be made available to editors and reviewers. We strongly encourage code deposition in a community repository (e.g. GitHub). See the Nature Portfolio guidelines for submitting code & software for further information.

Data

Policy information about availability of data

All manuscripts must include a data availability statement. This statement should provide the following information, where applicable:

- Accession codes, unique identifiers, or web links for publicly available datasets
- A description of any restrictions on data availability
- For clinical datasets or third party data, please ensure that the statement adheres to our policy

All sequencing data available under Accession no. SAMN35531338 - SAMN35530730 at the Sequence Read Archive (SRA) (http://www.ncbi.nlm.nih.gov/sra), and the associated BioProject is PRJNA978031. Reference Pf3D7 data available at https://plasmodb.org/plasmo/app and malaria incidence data available at https://data.malariaatlas.org. All de-identified datasets generated during the current study and used to make all figures are available as supplementary files or tables.

Research involving human participants, their data, or biological material

Policy information about studies with human participants or human data. See also policy information about sex, gender (identity/presentation), and sexual orientation and race, ethnicity and racism.

Reporting on sex and gender	Samples had been collected from rural areas in 12 districts as part of a large pfhrp2/3 deletion survey of those 12,572 study participants (56% male, 44% female, age ranges 0 and 99 years) presenting with clinical signs and symptoms of malaria.
Reporting on race, ethnicity, or other socially relevant groupings	We haven't used any socially relevant groupings in the current study.
Population characteristics	A total of 920 samples previously genotyped and MIP sequenced for pfhrp2/3 deletions from three regions of Ethiopia (Amhara = 598, Gambella = 83, Tigray = 239) (Supplementary Figure S1) were included in this analysis, representing dried blood spots taken from a subset of the overall series of 2637 malaria cases (Amhara = 1336, Gambella = 622, Tigray = 679) (Table S1).
Recruitment	Parent study was conducted to detect pfhrp2/pfhrp3 gene deletions in 3 regions in Ethiopia and used the WHO "Template protocols to support surveillance and research for pfhrp2/pfhrp3 gene deletions," available at https://www.who.int/malaria/publications/atoz/hrp2-deletion-protocol/en/. It was cross-sectional, multi site study in 11 districts along Ethiopia's borders with Eritrea, Sudan and South Sudan, located within three of its nine administrative regions. On average, ten health facilities were selected from each district, including four districts of Amhara Region (northwest Ethiopia), six districts of Tigray Region (north Ethiopia) and one district of Gambella region (southwest Ethiopia) during the 2017–2018 peak malaria transmission season (September–December, although enrolment in Gambella was completed in April 2018).
Ethics oversight	The parent study was approved by the Ethiopian Public Health Institute (Addis Ababa, Ethiopia; protocol EPHI-IRB-033-2017) and the World Health Organization Research Ethics Review Committee (Geneva, Switzerland; protocol ERC.0003174 001). Parasite sequencing and analysis of de-identified samples was deemed nonhuman subjects research by the University of North Carolina at Chapel Hill (NC, USA; study 17-0155).

Note that full information on the approval of the study protocol must also be provided in the manuscript.

Field-specific reporting

Please select the one below that is the best fit for your research. If you are not sure, read the appropriate sections before making your selection.

☒ Life sciences ☐ Behavioural & social sciences ☐ Ecological, evolutionary & environmental sciences

For a reference copy of the document with all sections, see nature.com/documents/nr-reporting-summary-flat.pdf

Life sciences study design

All studies must disclose on these points even when the disclosure is negative.

Sample size	Parent study used pfhrp2/3 deletions survey WHO protocol (https://www.who.int/publications/i/item/9789240002036) to select participants. Each facility passively enrolled participants presenting with symptoms of malaria (fever, headache, joint pain, feeling cold, nausea and/or poor appetite), with sample size proportionally allocated to each facility based on the previous year's malaria case load. For current study, we genotyped all available samples (a total of 920 samples previously genotyped for pfhrp2/3 deletions survey from three regions of Ethiopia (Amhara = 598, Gambella = 83, Tigray = 239) were further sequenced using two MIP panels; i) a drug resistance panel comprising 814 probes designed to target mutations and genes associated with antimalarial resistance and ii) a genome-wide SNP panel comprising 1832 probes). Unlike other epidemiological studies, sample size population genomic study is often ad hoc and the sample size > 50 per site is considered as good enough to capture all genomic metrics (https://journals.plos.org/plosgenetics/article?id=10.1371/journal.pgen.1007065).
Data exclusions	Samples with high missingness (>50%) removed (Extended Data Fig. 3), and total 609 samples and 1395 SNPs from the genome-wide panel (Extended Data Fig. 4, Supplementary Data 3 and 4) were included in downstream relatedness and PCA analyses. All resistance genotypes with sufficient depth and quality were included in downstream analysis.

Replication	For parent study all PCR assays were performed in duplicate. Deletion calls made by PCR were limited to samples with >100 parasites/µL, with negative pfhrp2 or pfhrp3 bands in both replicates, and positive by a final confirmatory real-time PCR assay. To increase confidence in pfhrp2/3 deletion calls, multiple confirmatory methods were employed, including PCR, MIP sequencing, WGS, and an HRP2 immunoassay.<br>In current experiment, we included positive control DNA (Pf3D7) to check our experiment is working and no DNA template to check contamination. Gele Image for PCR and Fragment analysis for library was checked by senior Lab technician and Postdoc in Lab for correct size of the MIP product before sequencing. Controlling false variant call and minimizing sequencing error is more important and thus, we used robust and optimize pipeline and more stringent filtering criteria as follows;<br>In current analysis, we used MIPWrangler software to stitch paired reads, remove sequence errors, and predict MIP microhaplotypes leveraging the unique molecular identifiers (UMIs) in each arm. Then only included loci with 10 UMI minimum count. Because dried blood spot sampling differed based on RDT results (participants with HRP2-/PfLDH+ results were purposefully oversampled for molecular characterization in the parent study), we adjusted K13 622I, and other key antimalarial drug resistance mutations prevalence estimate by weighting for the relative sampling proportions of RDT-concordant (HRP2+) and discordant (HRP2-/PfLDH+) samples.
Randomization	Parent study used WHO protocol, any subject presenting to study health facilities with symptoms of malaria was eligible for enrollment. Randomization was not performed in current data analysis as in current study we don't have pre experimental factors / categorical control variables that consider as known covariates that could affect our genotype results (mutations and deletion). However, we done most of our experiments such library preparation and sequencing in the same batch to minimize the batch effect (most common categorical control variables in omic experiments).
Blinding	In parent study, field staff were not blinded to malaria RDT results because they were used to inform clinical care according to national guidelines. Blinding was not performed in current data analysis. Blinding is very important to prevent to observer bias. In current study there is less chance of observer bias as we used known reference genome, optimized pipeline and analysis was done by expert on this area.

Reporting for specific materials, systems and methods

We require information from authors about some types of materials, experimental systems and methods used in many studies. Here, indicate whether each material, system or method listed is relevant to your study. If you are not sure if a list item applies to your research, read the appropriate section before selecting a response.

Materials & experimental systems

n/a	Involved in the study
☒	☐ Antibodies
☒	☐ Eukaryotic cell lines
☒	☐ Palaeontology and archaeology
☒	☐ Animals and other organisms
☒	☐ Clinical data
☒	☐ Dual use research of concern
☒	☐ Plants

Methods

n/a	Involved in the study
☒	☐ ChIP-seq
☒	☐ Flow cytometry
☒	☐ MRI-based neuroimaging
