# Supplementary material for: Plasmodium falciparum resistant to artemisinin and diagnostics have emerged in Ethiopia
Source: Nat Microbiol. 2023 Aug 28;8(10):1911–9. doi: 10.1038/s41564-023-01461-4 (PMC10522486; doi:10.1038/s41564-023-01461-4)

Brown University, USA

Bailey Lab Protocol
R. DeFeo
Version 1
220602
rebecca_defeo@brown.edu

Parasite (Pf) MIP Capture 96-well Protocol

This protocol is for performing parasite (Pf) MIP Captures on 96-well PCR plates. There are three main steps to MIP Captures: (1) MIP Capture (2) Exonuclease Treatment and (3) Pf MIP PCR. Each of these steps requires approximately 40-70 minutes on the thermal cycler, plus time for master mix preparation + dispensing.

If you cannot complete this whole protocol in one day, it is recommended that the protocol is completed through the Exonuclease Treatment - leaving the Pf MIP PCR for the next day. Also, because all of the thermal cycler programs end in a 4℃ hold step, it is OK to leave the machines running slightly over, without immediately beginning the next step.

Required Materials

- [ ] Molecular Grade Water (MGW)
- [ ] 1X TE Low EDTA Buffer
- [ ] Ampligase 100 U/µL
  Lucigen Cat # A0110K
- [ ] 1X Ampligase Storage Buffer
  - [ ] 1M Tris-Hcl, pH 7.5
    Invitrogen Cat # 15567027
  - [ ] 5M NaCl
    Sigma Cat # S5150
  - [ ] 500 mM EDTA
    Invitrogen Cat # AM9912
  - [ ] Triton X-100
    Sigma Cat # T8787
  - [ ] 1 M DTT (154.24 g/mol)
    Sigma # 10708984001
- [ ] 10X Ampligase Reaction Buffer
  Lucigen Cat # A1905B
- [ ] dNTP 10 mM
  NEB Cat # N0447L
- [ ] Phosphorylated MIP pools
- [ ] Q5 DNA Polymerase
  NEB Cat # M0491L
- [ ] Exo I
  NEB Cat # M0293L 20,000 U/mL
- [ ] Exo III
  NEB Cat # M0206L 100,000 U/mL
- [ ] 5X MMC
  - [ ] Ficoll 70
  - [ ] Ficoll 400
  - [ ] PVP360
- [ ] 5X Phusion Reaction Buffer
  NEB Cat # M0530L
- [ ] Phusion DNA Polymerase
  NEB Cat # M0530L
- [ ] Gel electrophoresis reagents and equipment
- [ ] Monarch® DNA Gel Extraction Kit
  NEB Cat # T1020

Before you start

- [ ] Phosphorylate balanced MIP pools.
- [ ] Make up 1X Ampligase Storage Buffer to prepare Ampligase 1 U/µL.

- [ ] Make up Molecular Mass Crowder mix.
- [ ] Create capture sheet
- [ ] Create MIP Pool (working concentration)

- [ ] Select the working FW and REV barcode plates [10 uM] that will be used in the PCR step of the MIP capture. These will be stored in -20C and frozen, so ensuring they are removed, placed at 4C and thawed by the time they are needed at the PCR step is essential for optimizing the time this protocol takes. See MIP Barcode Preparation for details of how to plate and dilute the barcode primers from stock plates [100 uM] to the working [10 uM] FW and REV barcode plates, if they are not already prepared. Keeping a record of the barcodes that are used on each MIP capture plate is mandatory for downstream sequencing + analysis.

Set Up

- [ ] Wipe down your bench and pipettes with 10% bleach. Let the bleach sit for a few minutes and then spray down your bench and pipettes with 70% ethanol, wait for ethanol to evaporate.
- [ ] Thaw reagents used in the 'capture' step as well as sample plates and control DNA on ice - *the reagents can take close to 45 minutes to thaw. Please do not expedite the thawing process by holding reagents in your hand.*

Capture sheet, barcodes and plate setup

Samples must be in LIST FORMAT for generating the capture sheet. The capture sheet must be created before you perform your captures, as it calculates the master mix volumes for you, based on the samples entered on the "sample_plates" tab.

Here is the template/example of a Pf MIP capture sheet. The boxes in green indicate "instructions" whereas the boxes in yellow indicate areas of "user-input fields" that are unique to the capture being performed.

The barcodes that are used in the Pf MIP PCR are to be recorded in the "sample_sheet" tab. It is important to select a unique barcode pair, to avoid barcode conflicts between several libraries for downstream sequencing.

Pf MIP Capture

- ☐ All sample plates, capture plates and controls are to be kept on cold blocks - switch out cold blocks if they are getting warm. All reagents are to be kept on ice and the tube used to prepare the master mix should also remain on ice.
- ☐ It is best practice to start the "MIP Capture 60" program on the thermal cycler before beginning, as the program preheats and begins with a "hold" temperature step.

**MIP Capture 60**	Lid: 100℃
95℃	HOLD - Pause to preheat
95℃	10 minutes
60℃	60 minutes
4℃	HOLD

- ☐ Vortex sample plates and centrifuge at 4000 rpm for 1 minute - samples extracted from chelex should be spun down for 5 minutes to ensure any residual chelex is pelleted to the bottom.
- ☐ Plate 5 uL of sample into each well on the capture plate.

  One important point to consider, before plating any sample and/or capture master mix onto your capture plate, is how much MIP capturing experience you have as a technician. If you are new to MIP captures, it is best practice to plate the samples first, ensuring to change tips with every row of samples. Once the samples are on the capture plate, prepare your capture master mix and add that to the capture plate, changing pipette tips with every row.

  There is an opportunity to use far less pipette tips by plating the capture master mix before the samples, though. The only concern to plating the master mix before plating the samples, is how long the master mix is sitting out - as these mixes perform most optimally, when they are mixed, plated and placed on the thermal cycler immediately after. As an experienced MIP capture technician, the sample plating process should not take longer than a couple of minutes, for one 96-well plate and if that is achievable, feel free to plate the master mix first, with one set of pipette tips and then add the samples to the capture plate, changing pipette tips with every row of samples.
- ☐ It is important to include a couple of positive controls - add 5 uL of control template to the plate.
- ☐ To create a Negative Capture Control (NTC) add 5 uL of MGW to one well.

Master Mix for **Pf MIP Capture**

	Volume (10 µL reaction)
MGW	0.84 µL
10X Ampligase Reaction Buffer	0.96 µL
5X MMC	2 µL
dNTP 10 mM (diluted 1:50)	0.2 µL
MIP pool (working concentration)	0.4 µL
Ampligase (1 U/µL)	0.4 µL
Q5 DNA Polymerase (diluted 1:50)	0.2 µL

- ☐ The MIP Capture Mastermix requires a 1:50 dilution of both Q5 DNA Polymerase and dNTP 10 mM. These reagents are diluted with a 1X Ampligase Reaction Buffer, which is made from the 10X Ampligase Reaction Buffer. **These dilutions must be made fresh for each set of MIP captures**. This mixture results in enough diluted products for 250 reactions, so duplicate this as necessary if you are doing multiple 96-well capture plates. Combine the following in a tube to make 1X Ampligase Reaction Buffer, vortex and spin down:

  10 uL **10X Ampligase Reaction Buffer** + 90 uL **MGW**
- ☐ Transfer 49 uL of the 1X Ampligase Reaction Buffer, into one new tube and another 49 uL into a second tube - one for Q5 and one for dNTP. Add 1 uL of dNTP 10 mM to one tube and 1 uL of Q5 DNA Polymerase to the second tube - the total volume in both of these tubes is now 50 uL. Vortex these tubes and spin down - this mixture is ready to use in the MIP Capture Mastermix.
- ☐ Prepare the MIP Capture Master Mix, per the Capture Sheet.

  It is important to **avoid** vortexing ANY enzymes throughout this process - mix the Ampligase 1 U/µL and the Q5 DNA Polymerase by flicking the tube.
- ☐ Once all reagents are combined to create the master mix, lightly vortex for just enough time to mix. Spin down the master mix tube so all droplets are off of the cap.
- ☐ Referring to the bottom left of the “master_mixes” tab, aliquot the designated volume of master mix into 12 PCR-strip tubes to allow for multichannel pipetting - keep this on a cold block.
- ☐ Using a multichannel, pipette 5 µL of master mix into each well - you *must* change pipette tips after each addition if the samples are already plated, as re-using a pipette tip will cause contamination. Don’t forget to add mastermix to your positive and negative control wells!
- ☐ Seal the plate, vortex briefly and then centrifuge for 1 minute @ 4000 rpm. Load the plate onto the thermocycler and continue the MIP Capture 60 program.
- ☐ At this point, return any reagents that will not be used for the Exonuclease Treatment or Pf MIP PCR steps, to the freezer. Gather the reagents that are required for the Exonuclease Treatment step and put them on ice.

Exonuclease Treatment

Master Mix for **Exonuclease Treatment**:

	**Volume (12 µL reaction)**
MGW	0.8 µL
10X Ampligase Reaction Buffer	0.2 µL
Exo I	0.5 µL
Exo III	0.5 µL

- ☐ All reagents are to be kept on ice. The tube used to prepare the master mix should also remain on ice. The capture plate should be on a cold block - switch out the cold block if it begins to get warm.
- ☐ Remove the capture plate from the thermal cycler and centrifuge for 1 minute @ 4000 rpm - ***do not vortex.***
- ☐ It is best practice to start the "Exo" program on the thermal cycler before beginning, as the program preheats and begins with a "hold" temperature step.

**Exo**	Lid: 100℃
37℃	HOLD
37℃	60 minutes
95℃	2 minutes
4℃	HOLD

- ☐ Prepare the Exonuclease Treatment Master Mix, per the Capture Sheet.
    - ☐ It is important to **avoid** vortexing ANY enzymes throughout this process - mix the Exo I and the Exo III by flicking the tube.
- ☐ Once all reagents are combined to create the master mix, lightly vortex for just enough time to mix. Spin down the master mix tube so all droplets are off of the cap.
- ☐ Referring to the bottom left of the "master_mixes" tab, aliquot the designated volume of master mix into 12 PCR-strip tubes to allow for multichannel pipetting - keep this on a cold block.
- ☐ Using a multichannel, pipette 2 µL of master mix into each well - you *must* change pipette tips after each addition, as the samples are already plated and re-using a pipette tip will cause contamination.
- ☐ Seal the plate, ***do not vortex,*** but centrifuge for 1 minute @ 4000 rpm. Load the plate onto the thermocycler and continue the Exo program.

- ☐ At this point, return any reagents that will not be used for the Pf MIP PCR step to the freezer. Gather the reagents that are required for the Pf MIP PCR step and put them on ice.

  If there is not enough time to begin the Pf MIP PCR step, once the Exo Program on the thermal cycler is complete, it is OK to place the MIP Capture plate in 4℃ overnight. The next day, continue with the Pf MIP PCR.

Pf MIP PCR

- ☐ Remove the capture plate from the thermal cycler and centrifuge for 1 minute @ 4000 rpm - ***do not vortex.***
- ☐ It is best practice to start the "Pf MIP PCR" program on the thermal cycler before beginning, as the program preheats and begins with a "hold" temperature step. The program will vary based on the number of cycles, but standard for Pf MIP PCR is 20 cycles.

**Pf MIP PCR 'n' Cycles**	Lid: 105℃	
98℃	HOLD	
98℃	30 seconds	
98℃	10 seconds	x 'n' cycles
63℃	30 seconds	
68℃	30 seconds	
68℃	2 minutes	
4℃	HOLD	

- ☐ First, add 12 uL of MGW to an empty well to create a Negative PCR Control (NTP).
- ☐ Before preparing the Pf MIP PCR Master Mix, add the FW and REV Barcodes to the capture plate - the barcodes act as a unique identifier for the sample, while also containing the primers for the PCR reaction. Each capture plate reaction gets 1.25 µL of FW barcodes and 1.25 µL REV barcodes. Don't forget to include your newly created, negative control well!

  Please note that the original well position of the barcodes and the 'destination' well position of those barcodes on the capture plate are assumed to be the same [in downstream sequencing]. For example; barcodes used on the Capture Plate in Row A must come from Row A on the FW barcode plate and Row A on the REV barcode plate. **If corresponding barcode well locations are not used properly, it must be recorded so accurate identifiers are used in the Illumina Nextseq run.**

Master Mix for **Pf MIP PCR**:

	**Volume (25.5 µL reaction)**
MGW	0.25
5X Phusion Reaction Buffer	5
5X MMC	5
10 mM dNTP	0.5
Phusion DNA Polymerase	0.25

- ☐ Prepare the Pf MIP PCR Master Mix, per the Capture Sheet.
  - ☐ It is important to **avoid** vortexing ANY enzymes throughout this process - mix the Phusion DNA Polymerase by flicking the tube.
- ☐ Once all reagents are combined to create the master mix, lightly vortex for just enough time to mix. Spin down the master mix tube so all droplets are off of the cap.
- ☐ Referring to the bottom left of the "master_mixes" tab, aliquot the designated volume of master mix into 12 PCR-strip tubes to allow for multichannel pipetting - keep this on a cold block.
- ☐ Using a multichannel, pipette 11 µL of master mix into each well - you *must* change pipette tips after each addition, as the samples are already plated and re-using a pipette tip will cause contamination. Don't forget your negative control well!
- ☐ Seal the plate, ***do not vortex,*** but centrifuge for 1 minute @ 4000 rpm. Load the plate onto the thermocycler and continue the Pf MIP PCR program.
- ☐ At this point, return all reagents and barcode plates to the freezer.
- ☐ Once the PCR program is finished, remove the plate from the thermal cycler and bring it to a 4℃ Post-PCR refrigerator.

Gel electrophoresis

It is best practice to 'gel-check' every capture plate to ensure that there are in fact successful captures, before moving on. It is especially important if a newly created/phosphorylated MIP pool was used for the captures, as there is a chance it could not perform properly.

To avoid wasting (precious) capture volume, ALWAYS gel-check **<u>all</u>** of the positive and negative controls - it is up to the individual whether or not to also check a few samples, at random.

- [ ] Prepare a 1.5% agarose gel and 0.5X TBE Buffer
    - [ ] Small gel (7 cm x 8 cm, max. 20 wells) = 0.6g Agarose + 40mL 0.5X TBE Buffer
    - [ ] Medium gel (12 cm x 14 cm, max. 40 wells) = 1.2 g Agarose + 80 mL 0.5X TBE Buffer
    - [ ] Large gel (23 cm x 14 cm, max. 150 wells) = 2. 4 g Agarose + 160 mL 0.5X TBE Buffer
- [ ] Mix 1.3 µL of (SYBR Gold) loading dye with 5 µL of MIP Capture Product

    Be sure to aliquot the loading dye for the gel in the main lab and walk it over along with the MIP Capture Plate to Post-PCR and combine them there - NO Post-PCR MIP Captures should be opened in the main lab.
- [ ] Load the full 6.3 µL into the wells
- [ ] Run the gel
    - [ ] Small gel: 120V for 40 minutes
    - [ ] Medium gel: 200V for 40 minutes
    - [ ] Large gel: 135V for 45 minutes
- [ ] Image the gel
    - Be sure to take a low exposure and a high exposure image.
    - Export to jpeg
    - Maximize the size of the image before exporting
    - Quality = 100%

Pooling and 1st clean up

- [ ] Pool 2-5µL of each sample with a multi-channel pipette, including all positive and negative controls, dispensing into 12 strip-PCR tubes - be sure to change pipette tips between every row of samples. Combine and transfer the total volume in the strip tubes to an 1.5 or 2 mL eppendorf tube.

  NOTE: If your total volume of pooled captures is > 900 uL the clean up will need to be done in a tube greater than 2 mL (Bailey lab has 5 mL and 15 mL tube options and a magnet that can hold these tubes).
- [ ] Vortex briefly to mix and centrifuge at 16000 x g for 5 minutes.
- [ ] Transfer the top 90% of the volume to a new tube, avoiding the pellet of debris that settled to the bottom of the tube. Be sure to double check your final volume before adding beads, as pipette error and the spin down typically results in lesser volume than anticipated.

  NOTE: Another option is to do a partial clean up, for example; the total volume of pooled captures is 10 mL. Feel free to take 2-4 mL of this pool, spin it down, transfer to a new tube, avoiding debris at the bottom and clean just that portion of the pool. The remaining volume that was not spun down to be cleaned can be saved.
- [ ] Following the “Pooling and Cleanup” tab of the Capture sheet, mix the pooled product with 1.2x volume of DIY bead solution (Ampure XP A63881, SPRI, or equivalent) by inverting 10 times. Briefly spin down the tube to pull droplets off of the cap.
- [ ] Let the pool and the beads incubate at room temperature for 10 minutes.
- [ ] Put the tubes on the magnet and wait for the solution to clear as the beads move to the magnetized wall of the tube - about 10 minutes.
- [ ] Remove the supernatant and transfer it to a new tube, which will be labeled “[insert library name] FT” (i.e. 220504 SPDIV DR2 FT). FT = Flow Through and we reserve this in case the DNA did not get pulled by the magnetic beads; once a gel has been run, to confirm DNA is present, then it can be discarded.
- [ ] Without disturbing the pellet, wash the beads with freshly made 80% ethanol - enough to cover the top of the beads. Let the wash sit for 30 seconds and then remove and dispose of the ethanol.
- [ ] Repeat the previous wash step.
- [ ] After removing the ethanol of the second wash, allow the beads to dry on the magnet for 5-10 minutes, until the ethanol evaporates. 5 minutes for 1.5/2 mL bead cleans , 7-10 minutes for 5+ mL bead cleans. Be sure to remove any residual ethanol that collects at the bottom of the tube during this time, so that it does not “rehydrate” the beads at the very bottom.

- ☐ Remove from the magnet and elute in 1X TE low EDTA - the volume of elution varies, based on the "Pooling and Cleanup" tab of the Capture Sheet. This sheet calculates the elution to be 10% of the initial pooled volume so if your MIP pool product was 500 uL, the elution will be 50 uL.

  NOTE: If you are afraid that your MIPs did not capture well and you want to concentrate your MIP pool Library down as much as possible, consider lowering the elution to 5% of the input volume. If you have a pool that was between 200-350 uL, you may consider eluting in 20 uL because that is what is required for the gel excision.

  Any MIP pool product that was less than 200 uL will still be eluted in 20 uL as that is the required volume for gel excision.
- ☐ Gently flick the tube to resuspend the beads off of the tube wall and incubate for 10 mins at room temperature.
- ☐ Place the tube back on the magnet until the solution is clear, about 5 minutes, and transfer the liquid to a new tube. This is your cleaned library, aka a "1.2x product", which can be labeled "[insert library name] 1.2x" (i.e. 220504 SPDIV DR2 1.2x).
- ☐ Optional: Run 5 µL of the 1.2x library on a gel to check for product. The goal of this gel-check is to ensure that the pool will show a visible band at the appropriate size, per the MIP Pool used in the captures. NOTE: 20 µL of 1.2x library is required for gel excision, so if you have ≤ 20 µL it is best to go straight to the gel extraction step.

Gel Extraction

- ☐ Gel extractions are done on the smallest gel, with the widest comb available (i.e. 7 cm x 8 cm gel, 6 spot comb). Prepare a 1.5% agarose gel using 0.5x TBE Buffer. For short MIP panels (final amplicon size less than 250bp) run a 2% gel.
- ☐ For the library to be extracted, mix 20 µL of the "1.2x product" with 5 µL of loading dye.
  Any leftover 1.2x product can be saved and revisited later for more excisions, if necessary.

  Prepared Loading Dye is made with the following:

SYBR GOLD	6 µL
MGW	194 µL
6X orange Loading Dye	1 mL

- ☐ For the ladder, mix 5 µL of loading dye, 5 µL of 1kb ladder, and 15 µL of MGW.

- ☐ The gel should run at 120V for 70 minutes. (90 minutes for short MIP panels, mentioned above).
- ☐ After the run is complete, place the gel on the bluelight imager and cut out the desired band, transferring the gel slice into a 1.5 mL or 2 mL eppendorf tube. Note the brightness/intensity of the band that was excised determines the amount of elution in the purification step.
  Common Pf MIP Capture Band Sizes:

DR2 (+ HAP, if applicable)	400 bp
IBC	300 bp
HeOME96	
DR3	

- ☐ The following instructions apply to the **NEB DNA Gel Extraction Kit T1020S.** If a different kit is being used, follow the instructions of the extraction kit.
- ☐ Weigh the gel slice by zero-ing the balance with the same type of tube that the gel slice is in and then placing the tube with the gel slice on the balance.
- ☐ Add the appropriate volume of gel dissolving buffer by taking the weight of the gel in mg and multiply 4X - that is the number of µL to add.
  i.e. if the gel slice is 180 mg, add 720 µL of gel dissolving buffer.
- ☐ Once the buffer is added, vortexing for several minutes can expedite the dissolving process - or feel free to leave it alone at RT, vortexing every 5-10 minutes, until there is no visible signs of gel.
  - ☐ The T1020S kit suggests using heat, but the probes are better preserved without.
- ☐ When the gel is completely dissolved, transfer no more than 750 µL to a spin column, inserted into a collection tube.
- ☐ Centrifuge at 16000 x g for 1 minute (Bailey Lab Program #1) and discard the FT. Place the column back into the collection tube.
  - ☐ IF there is more volume of dissolved gel mixture remaining, after you discard the supernatant from the first spin, place the column back in the collection tube, and add another 750 uL to the *same* spin column and repeat until all of the dissolved gel mixture passes through the column. Ensure that all FT is discarded and the column is placed back into the collection tube before moving on.
- ☐ Add 200 µL of wash buffer to the spin column and centrifuge at 16000 x g for 1 minute.
- ☐ Repeat for a total of 2 washes.

- [ ] Transfer the spin column to a clean 1.5 mL microcentrifuge tube and discard the collection tube.
- [ ] Pipette elution buffer (EB) directly onto the filter of the column and let incubate at room temperature for 1 minute.

Weak (dim) band	6-8 uL EB
Average	10-12 uL EB
Strong (bright) band	15-20 uL EB

- [ ] Spin down at 16000 x g for 1 minute and transfer the final elution to an o-ring, screw cap tube - this aids in preserving the library for multiple sequencing runs. This product is the library of the sample set that was captured (i.e. 220504 SPDIV DR2 Lib).

Quality Check

- [ ] To quantify the DNA content of the library, Qubit is an option. The typical range of libraries can vary greatly, but we hope that it is ~ 1 ng/uL as that is the concentration required when pooling for a Nextseq run. Libraries with concentrations less than 1 ng/uL have also sequenced just fine.
- [ ] Libraries are then sent for Fragment Analyzer to ensure that the library is pure. Transfer 2 uL of the final library to a snaptop 1.5 mL centrifuge tube labeled with a shortened title for your library and your initials (i.e. HSU R.C). If the library qubit more than 2 ng/uL it is best to dilute it down to ~ 1 ng/uL not only to best reflect the sequencing conditions, but to conserve library volume. Any library greater than 6 ng/uL ***must*** be diluted, per FA parameters.
- [ ] If there are unexpected peaks in the FA results, a new gel extraction can be done or further clean-up steps may be necessary.

FA Results

Ideally, FA results will look something like this, where the only peak is at the size of the product of interest:

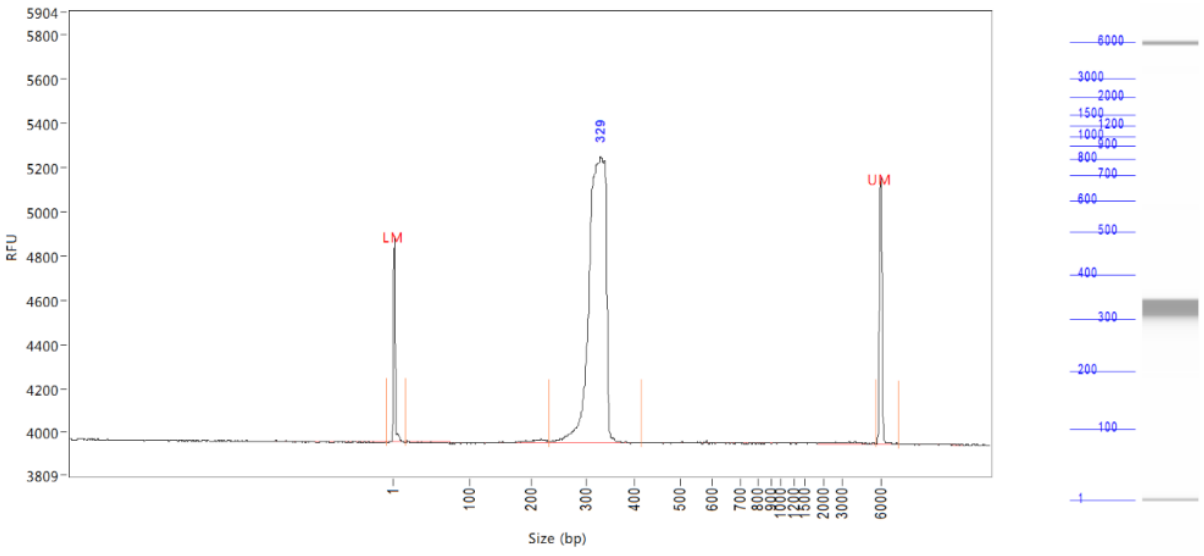

Sometimes, they will look like this, where a secondary peak at ~200-250 bp is present - this is OK, as that represents self-ligated probe, but know that the library is not as pure as the one above:

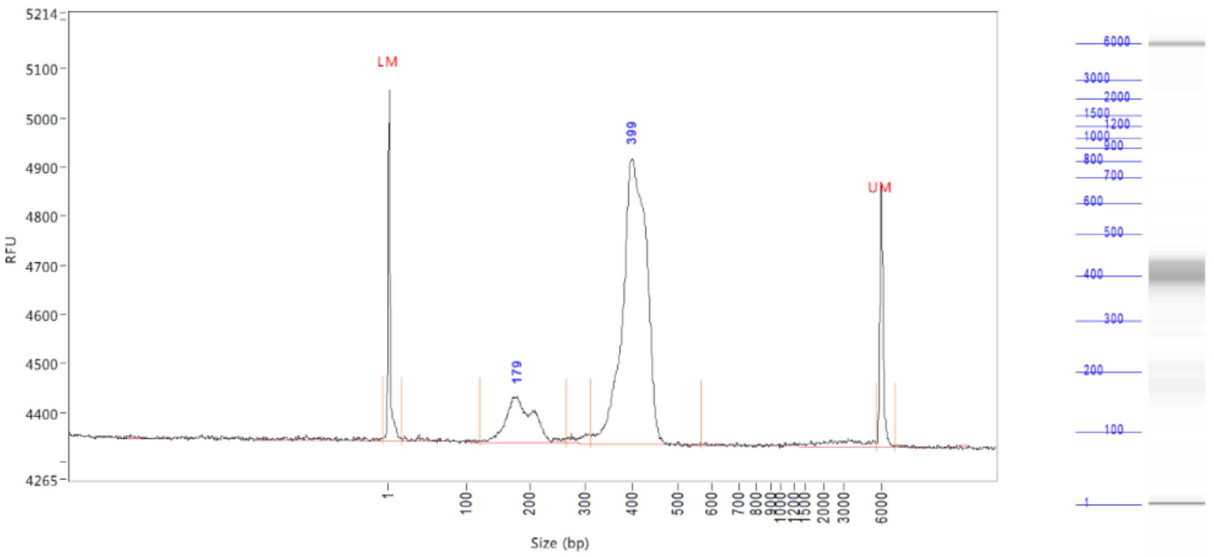

Supplement: Supplementary file 6 — Parasite (Pf) MIP capture 96-well protocol. [file 41564_2023_1461_MOESM6_ESM.pdf]
